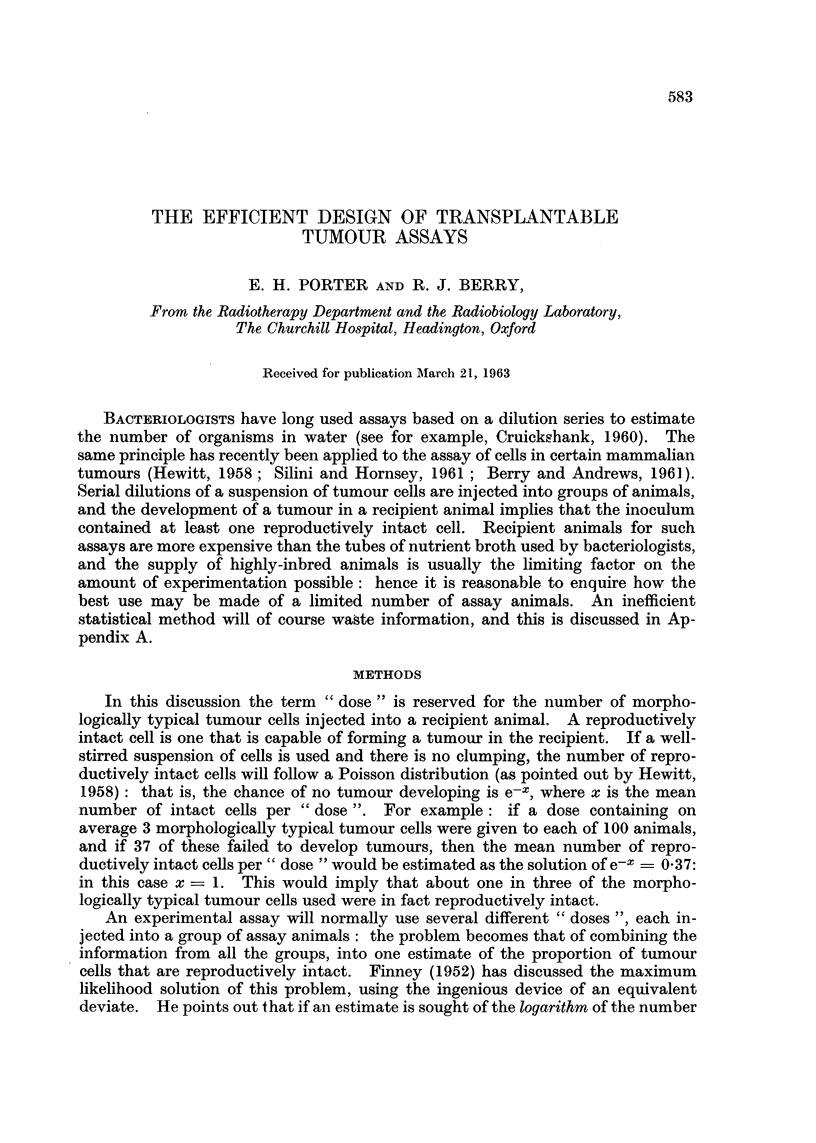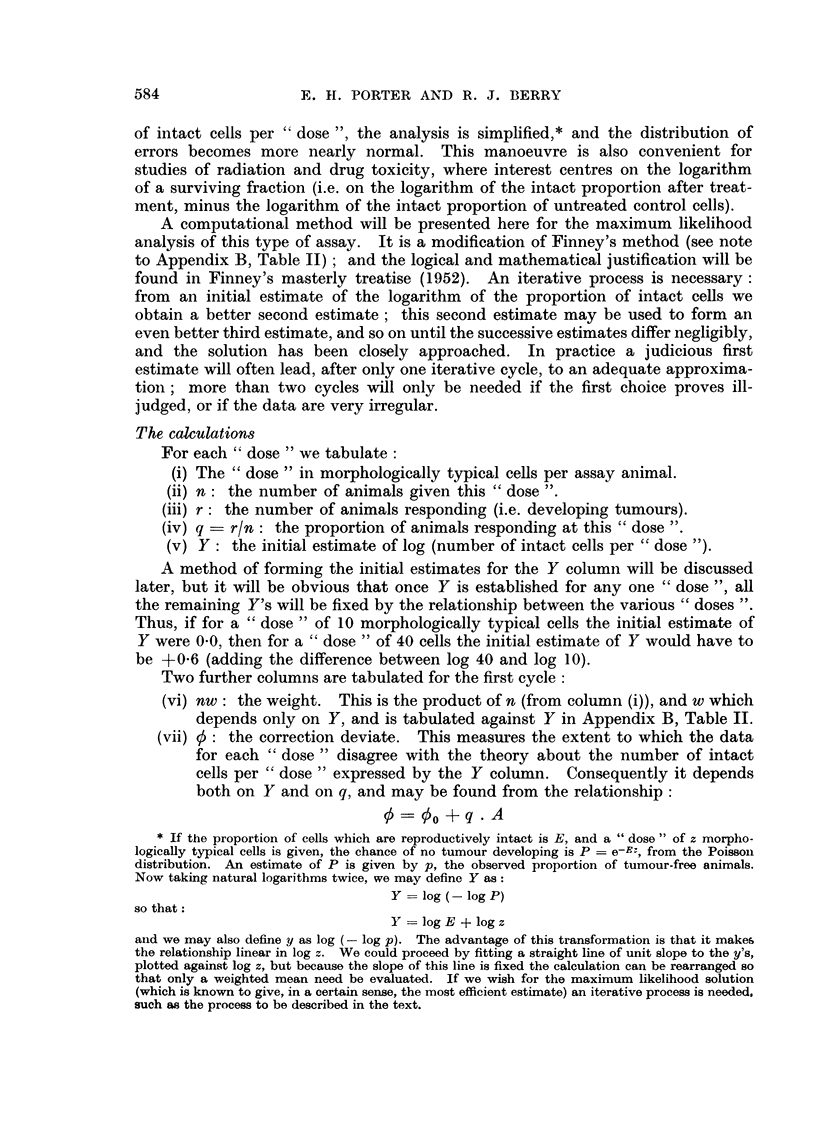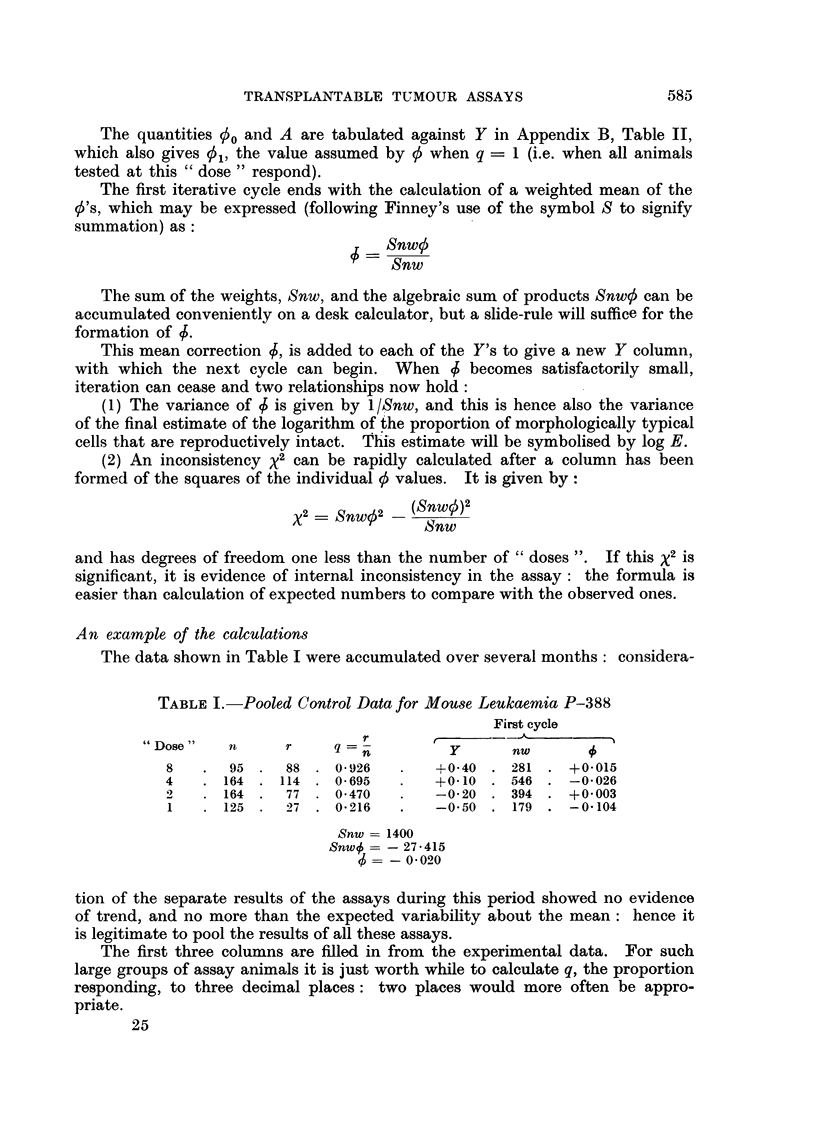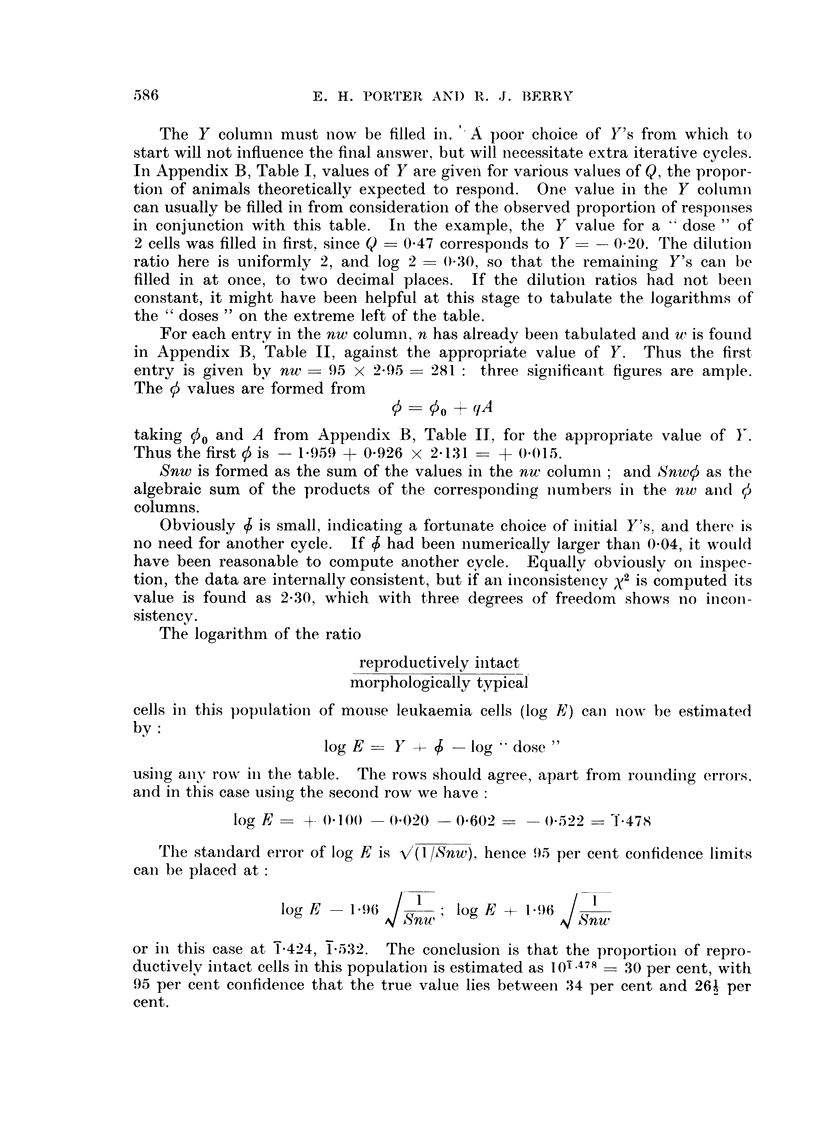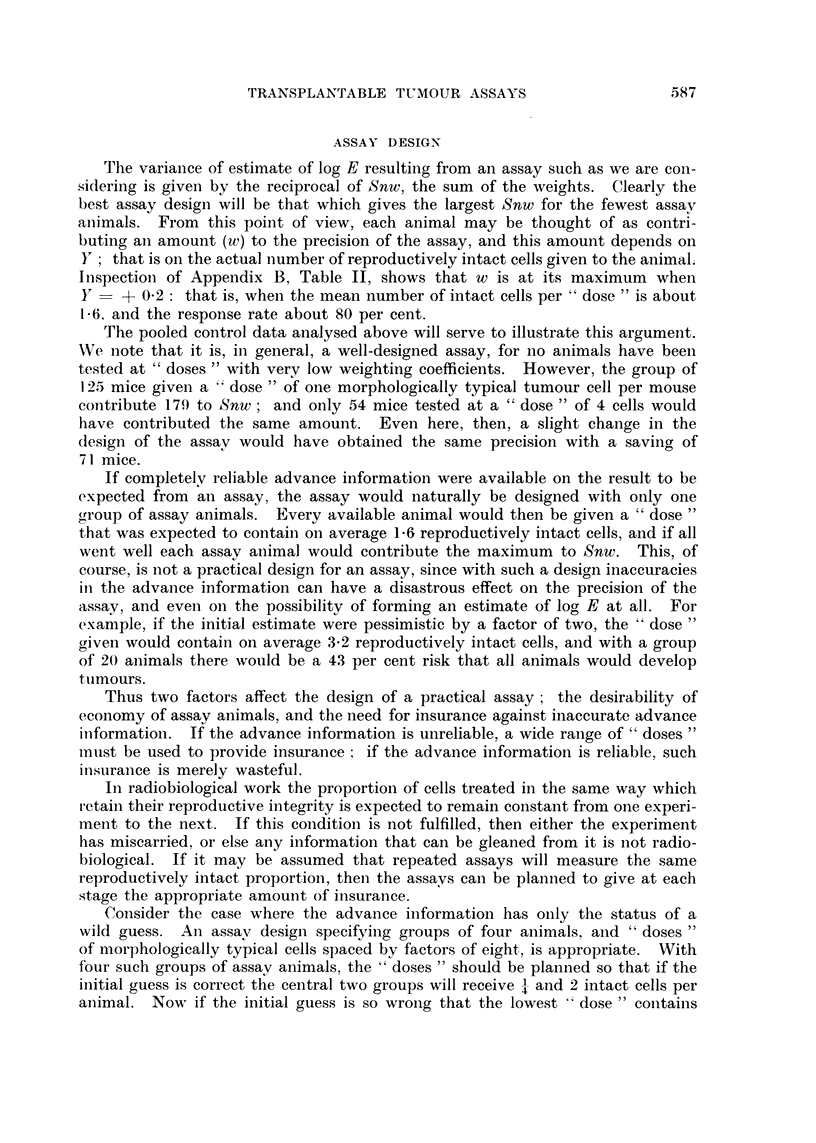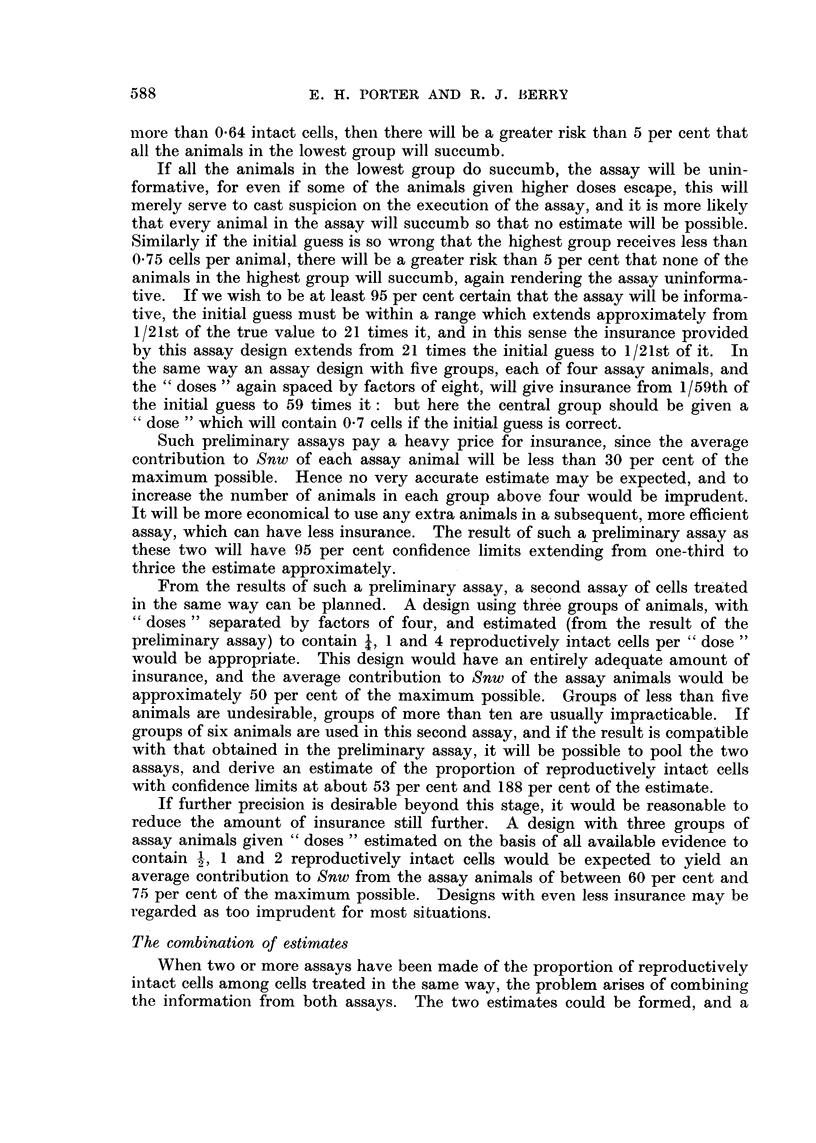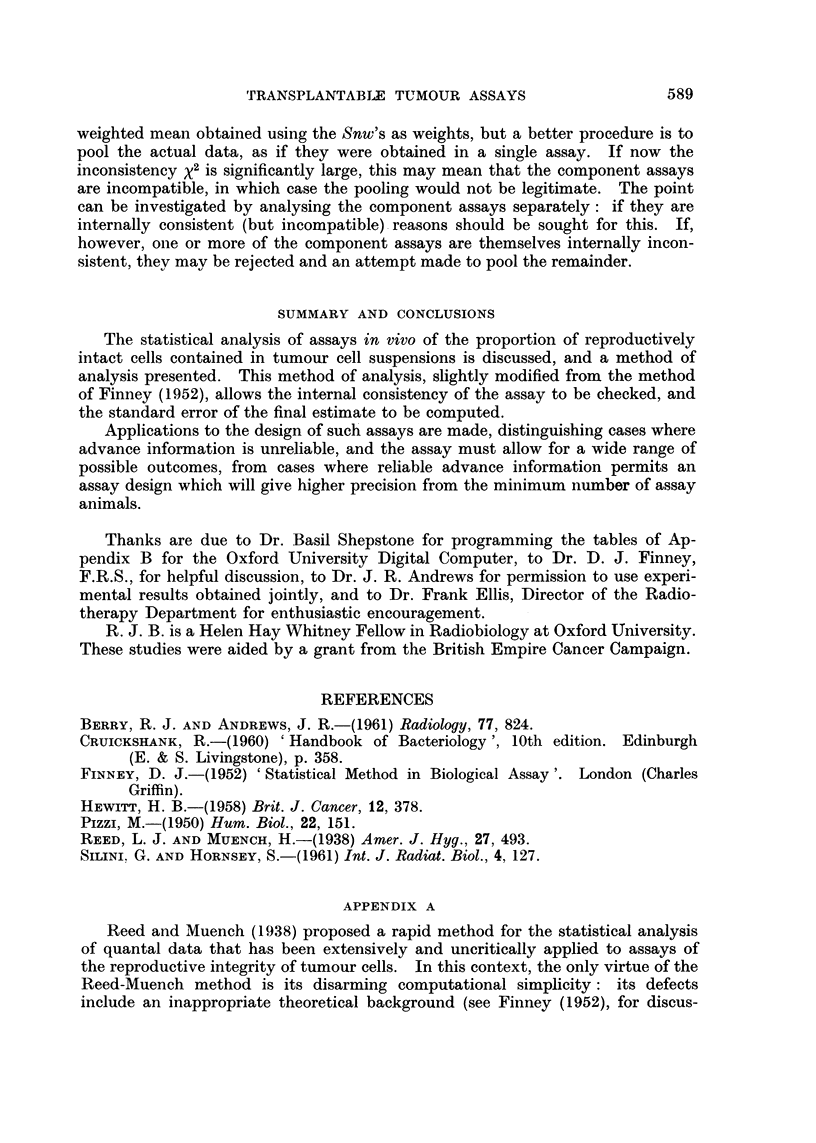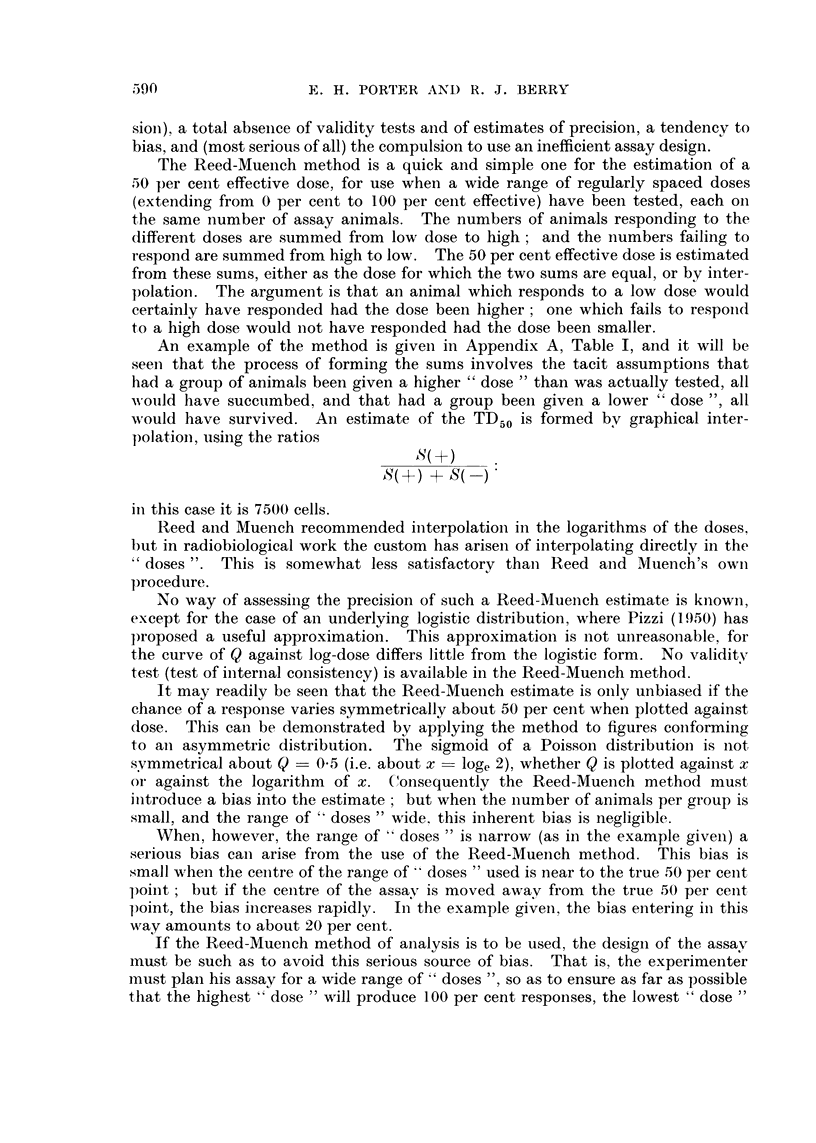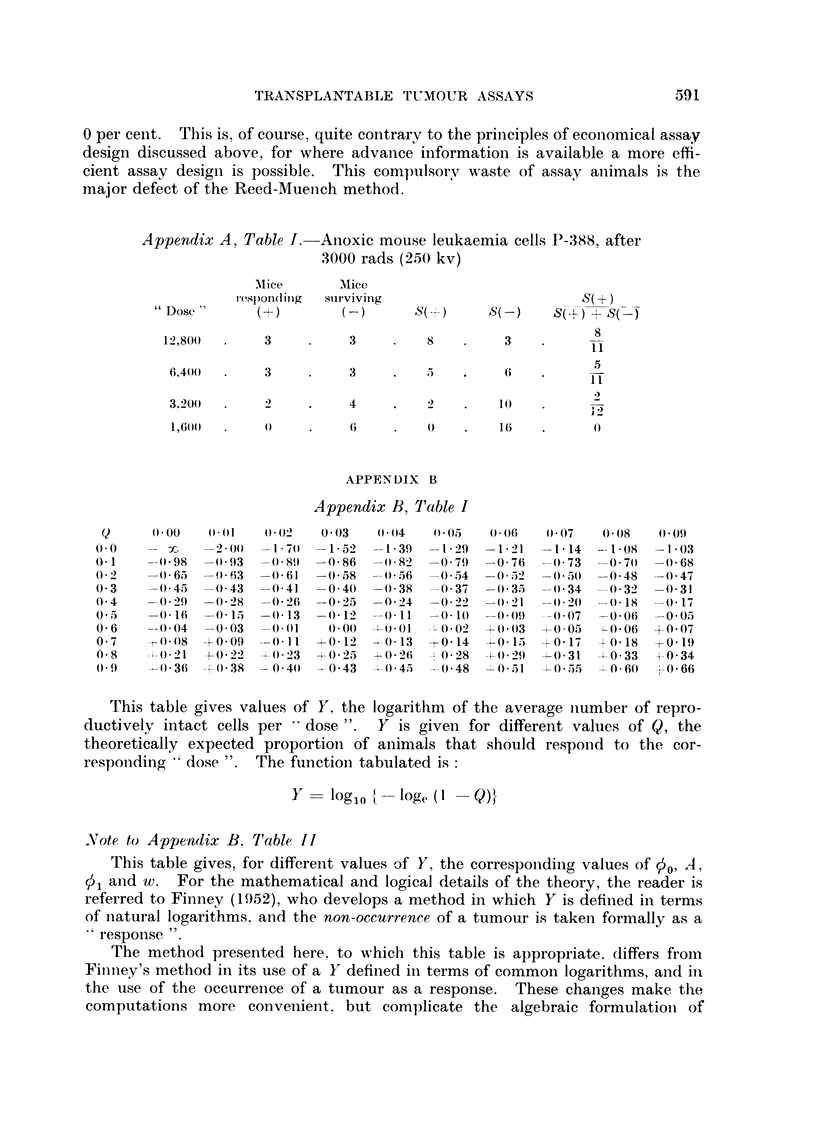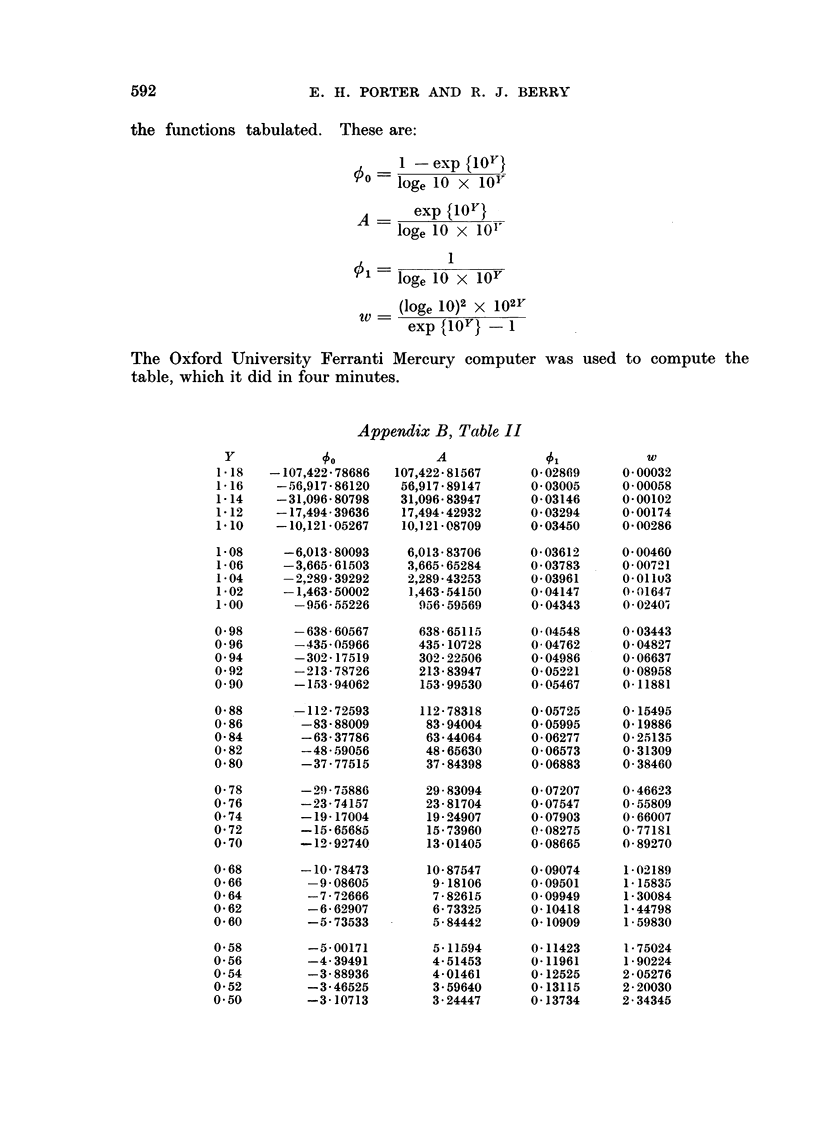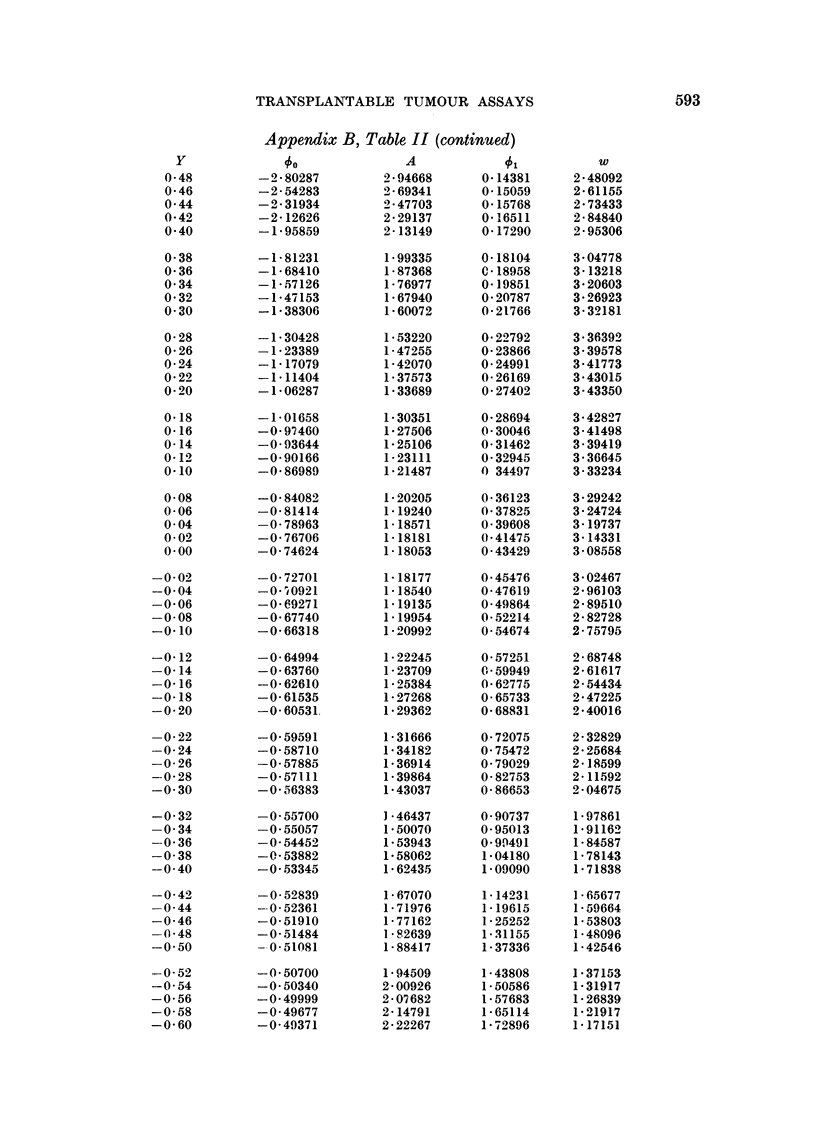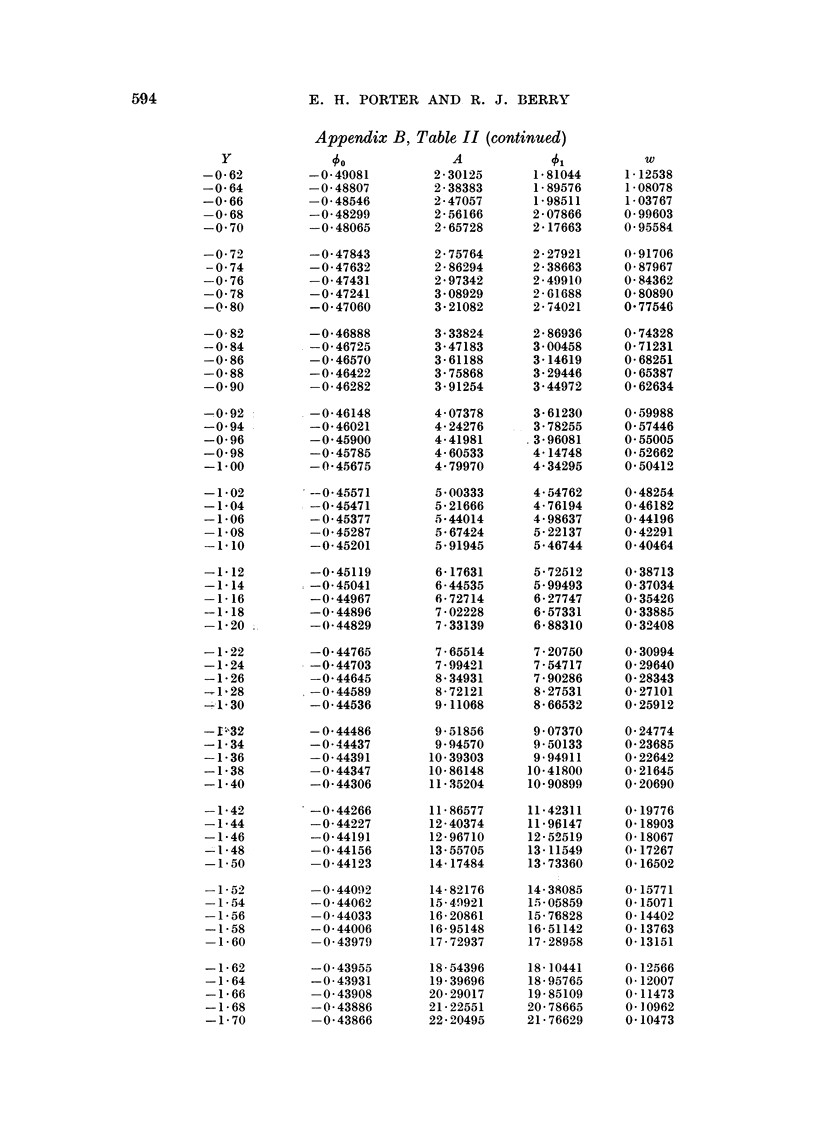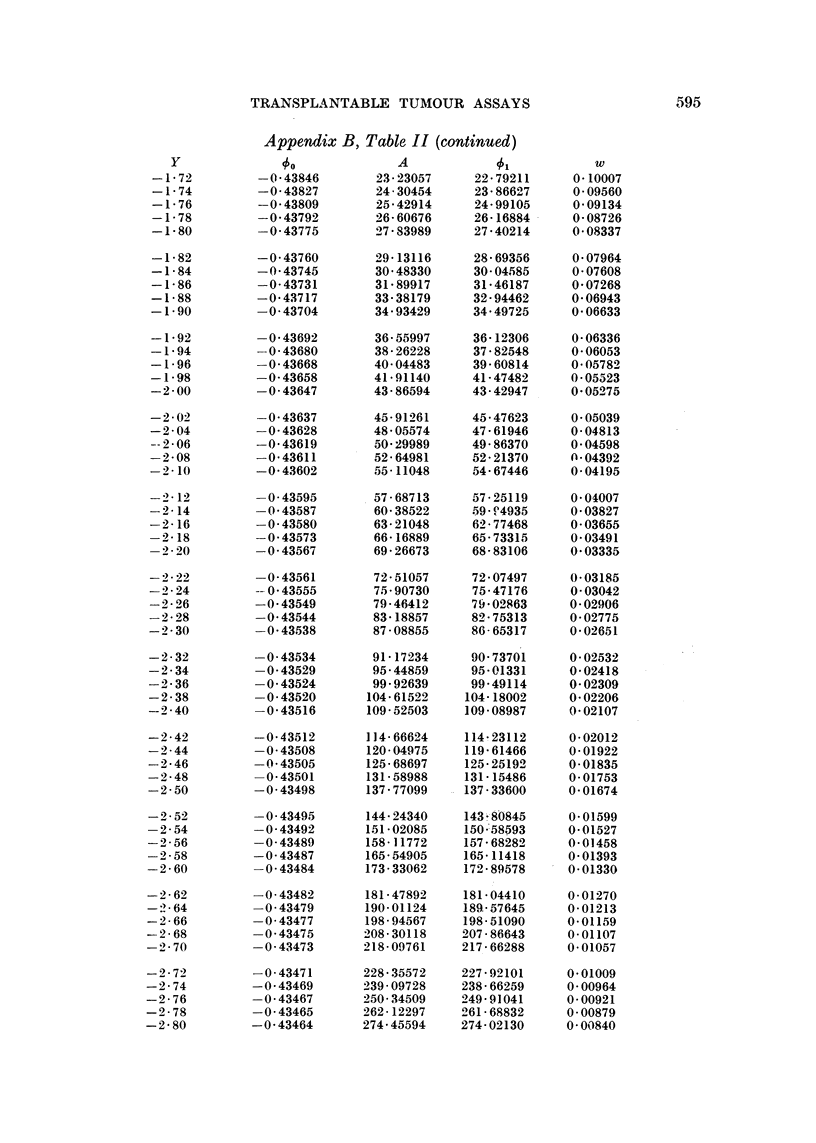# The Efficient Design of Transplantable Tumour Assays

**DOI:** 10.1038/bjc.1963.78

**Published:** 1963-12

**Authors:** E. H. Porter, R. J. Berry


### THE EFFICIENT DESIGN OF TRANSPLANTABLE TUMOUR ASSAYS

E. H. PORTER AND R. J. BERRY,

*From the Radiotherapy Department and the Radiobiology Laboratory, The Churchill Hospital, Headington, Oxford*

Received for publication March 21, 1963

BACTERIOLOGISTS have long used assays based on a dilution series to estimate the number of organisms in water (see for example, Cruickshank, 1960). The same principle has recently been applied to the assay of cells in certain mammalian tumours (Hewitt, 1958 ; Silini and Hornsey, 1961 ; Berry and Andrews, 1961). Serial dilutions of a suspension of tumour cells are injected into groups of animals, and the development of a tumour in a recipient animal implies that the inoculum contained at least one reproductively intact cell. Recipient animals for such assays are more expensive than the tubes of nutrient broth used by bacteriologists, and the supply of highly-inbred animals is usually the limiting factor on the amount of experimentation possible : hence it is reasonable to enquire how the best use may be made of a limited number of assay animals. An inefficient statistical method will of course waste information, and this is discussed in Appendix A.

### METHODS

In this discussion the term " dose " is reserved for the number of morphologically typical tumour cells injected into a recipient animal. A reproductively intact cell is one that is capable of forming a tumour in the recipient. If a well-stirred suspension of cells is used and there is no clumping, the number of reproductively intact cells will follow a Poisson distribution (as pointed out by Hewitt, 1958) : that is, the chance of no tumour developing is $e^{-x}$, where $x$ is the mean number of intact cells per " dose ". For example : if a dose containing on average 3 morphologically typical tumour cells were given to each of 100 animals, and if 37 of these failed to develop tumours, then the mean number of reproductively intact cells per " dose " would be estimated as the solution of $e^{-x} = 0{\cdot}37$: in this case $x = 1$. This would imply that about one in three of the morphologically typical tumour cells used were in fact reproductively intact.

An experimental assay will normally use several different " doses ", each injected into a group of assay animals : the problem becomes that of combining the information from all the groups, into one estimate of the proportion of tumour cells that are reproductively intact. Finney (1952) has discussed the maximum likelihood solution of this problem, using the ingenious device of an equivalent deviate. He points out that if an estimate is sought of the *logarithm* of the number of intact cells per " dose ", the analysis is simplified,* and the distribution of errors becomes more nearly normal. This manoeuvre is also convenient for studies of radiation and drug toxicity, where interest centres on the logarithm of a surviving fraction (i.e. on the logarithm of the intact proportion after treatment, minus the logarithm of the intact proportion of untreated control cells).

A computational method will be presented here for the maximum likelihood analysis of this type of assay. It is a modification of Finney's method (see note to Appendix B, Table II) ; and the logical and mathematical justification will be found in Finney's masterly treatise (1952). An iterative process is necessary : from an initial estimate of the logarithm of the proportion of intact cells we obtain a better second estimate ; this second estimate may be used to form an even better third estimate, and so on until the successive estimates differ negligibly, and the solution has been closely approached. In practice a judicious first estimate will often lead, after only one iterative cycle, to an adequate approximation ; more than two cycles will only be needed if the first choice proves ill-judged, or if the data are very irregular.

*The calculations*

For each " dose " we tabulate :

(i) The " dose " in morphologically typical cells per assay animal.
(ii) $n$ : the number of animals given this " dose ".
(iii) $r$ : the number of animals responding (i.e. developing tumours).
(iv) $q = r/n$ : the proportion of animals responding at this " dose ".
(v) $Y$ : the initial estimate of log (number of intact cells per " dose ").

A method of forming the initial estimates for the $Y$ column will be discussed later, but it will be obvious that once $Y$ is established for any one " dose ", all the remaining $Y$'s will be fixed by the relationship between the various " doses ". Thus, if for a " dose " of 10 morphologically typical cells the initial estimate of $Y$ were 0·0, then for a " dose " of 40 cells the initial estimate of $Y$ would have to be +0·6 (adding the difference between log 40 and log 10).

Two further columns are tabulated for the first cycle :

(vi) $nw$ : the weight. This is the product of $n$ (from column (i)), and $w$ which depends only on $Y$, and is tabulated against $Y$ in Appendix B, Table II.
(vii) $\phi$ : the correction deviate. This measures the extent to which the data for each " dose " disagree with the theory about the number of intact cells per " dose " expressed by the $Y$ column. Consequently it depends both on $Y$ and on $q$, and may be found from the relationship :

$$\phi = \phi_0 + q \cdot A$$

* If the proportion of cells which are reproductively intact is $E$, and a " dose " of $z$ morphologically typical cells is given, the chance of no tumour developing is $P = e^{-Ez}$, from the Poisson distribution. An estimate of $P$ is given by $p$, the observed proportion of tumour-free animals. Now taking natural logarithms twice, we may define $Y$ as :

$$Y = \log (- \log P)$$

so that :

$$Y = \log E + \log z$$

and we may also define $y$ as log (— log $p$). The advantage of this transformation is that it makes the relationship linear in log $z$. We could proceed by fitting a straight line of unit slope to the $y$'s, plotted against log $z$, but because the slope of this line is fixed the calculation can be rearranged so that only a weighted mean need be evaluated. If we wish for the maximum likelihood solution (which is known to give, in a certain sense, the most efficient estimate) an iterative process is needed, such as the process to be described in the text.

The quantities $\phi_0$ and $A$ are tabulated against $Y$ in Appendix B, Table II, which also gives $\phi_1$, the value assumed by $\phi$ when $q = 1$ (i.e. when all animals tested at this " dose " respond).

The first iterative cycle ends with the calculation of a weighted mean of the $\phi$'s, which may be expressed (following Finney's use of the symbol $S$ to signify summation) as :

$$\bar{\phi} = \frac{Snw\phi}{Snw}$$

The sum of the weights, $Snw$, and the algebraic sum of products $Snw\phi$ can be accumulated conveniently on a desk calculator, but a slide-rule will suffice for the formation of $\bar{\phi}$.

This mean correction $\bar{\phi}$, is added to each of the $Y$'s to give a new $Y$ column, with which the next cycle can begin. When $\bar{\phi}$ becomes satisfactorily small, iteration can cease and two relationships now hold :

(1) The variance of $\bar{\phi}$ is given by $1/Snw$, and this is hence also the variance of the final estimate of the logarithm of the proportion of morphologically typical cells that are reproductively intact. This estimate will be symbolised by log $E$.

(2) An inconsistency $\chi^2$ can be rapidly calculated after a column has been formed of the squares of the individual $\phi$ values. It is given by :

$$\chi^2 = Snw\phi^2 - \frac{(Snw\phi)^2}{Snw}$$

and has degrees of freedom one less than the number of " doses ". If this $\chi^2$ is significant, it is evidence of internal inconsistency in the assay : the formula is easier than calculation of expected numbers to compare with the observed ones.

*An example of the calculations*

The data shown in Table I were accumulated over several months : considera-

TABLE I.—*Pooled Control Data for Mouse Leukaemia P–388*

| " Dose " | $n$ | $r$ | $q = \frac{r}{n}$ | First cycle $Y$ | $nw$ | $\phi$ |
|---|---|---|---|---|---|---|
| 8 | 95 | 88 | 0·926 | +0·40 | 281 | +0·015 |
| 4 | 164 | 114 | 0·695 | +0·10 | 546 | −0·026 |
| 2 | 164 | 77 | 0·470 | −0·20 | 394 | +0·003 |
| 1 | 125 | 27 | 0·216 | −0·50 | 179 | −0·104 |

$Snw = 1400$
$Snw\phi = -27\cdot415$
$\bar{\phi} = -0\cdot020$

tion of the separate results of the assays during this period showed no evidence of trend, and no more than the expected variability about the mean : hence it is legitimate to pool the results of all these assays.

The first three columns are filled in from the experimental data. For such large groups of assay animals it is just worth while to calculate $q$, the proportion responding, to three decimal places : two places would more often be appropriate.

The $Y$ column must now be filled in. A poor choice of $Y$'s from which to start will not influence the final answer, but will necessitate extra iterative cycles. In Appendix B, Table I, values of $Y$ are given for various values of $Q$, the proportion of animals theoretically expected to respond. One value in the $Y$ column can usually be filled in from consideration of the observed proportion of responses in conjunction with this table. In the example, the $Y$ value for a " dose " of 2 cells was filled in first, since $Q = 0{\cdot}47$ corresponds to $Y = -0{\cdot}20$. The dilution ratio here is uniformly 2, and log 2 = 0·30, so that the remaining $Y$'s can be filled in at once, to two decimal places. If the dilution ratios had not been constant, it might have been helpful at this stage to tabulate the logarithms of the " doses " on the extreme left of the table.

For each entry in the $nw$ column, $n$ has already been tabulated and $w$ is found in Appendix B, Table II, against the appropriate value of $Y$. Thus the first entry is given by $nw = 95 \times 2{\cdot}95 = 281$ : three significant figures are ample. The $\phi$ values are formed from

$$\phi = \phi_0 + qA$$

taking $\phi_0$ and $A$ from Appendix B, Table II, for the appropriate value of $Y$. Thus the first $\phi$ is $-1{\cdot}959 + 0{\cdot}926 \times 2{\cdot}131 = +0{\cdot}015$.

$Snw$ is formed as the sum of the values in the $nw$ column ; and $Snw\phi$ as the algebraic sum of the products of the corresponding numbers in the $nw$ and $\phi$ columns.

Obviously $\bar{\phi}$ is small, indicating a fortunate choice of initial $Y$'s, and there is no need for another cycle. If $\bar{\phi}$ had been numerically larger than 0·04, it would have been reasonable to compute another cycle. Equally obviously on inspection, the data are internally consistent, but if an inconsistency $\chi^2$ is computed its value is found as 2·30, which with three degrees of freedom shows no inconsistency.

The logarithm of the ratio

$$\frac{\text{reproductively intact}}{\text{morphologically typical}}$$

cells in this population of mouse leukaemia cells (log $E$) can now be estimated by :

$$\log E = Y + \bar{\phi} - \log \text{" dose "}$$

using any row in the table. The rows should agree, apart from rounding errors, and in this case using the second row we have :

$$\log E = +0{\cdot}100 - 0{\cdot}020 - 0{\cdot}602 = -0{\cdot}522 = \bar{1}{\cdot}478$$

The standard error of log $E$ is $\sqrt{(1/Snw)}$, hence 95 per cent confidence limits can be placed at :

$$\log E - 1{\cdot}96\sqrt{\frac{1}{Snw}}\ ;\ \log E + 1{\cdot}96\sqrt{\frac{1}{Snw}}$$

or in this case at $\bar{1}{\cdot}424$, $\bar{1}{\cdot}532$. The conclusion is that the proportion of reproductively intact cells in this population is estimated as $10^{\bar{1}\cdot478} = 30$ per cent, with 95 per cent confidence that the true value lies between 34 per cent and 26½ per cent.

### ASSAY DESIGN

The variance of estimate of log $E$ resulting from an assay such as we are considering is given by the reciprocal of $Snw$, the sum of the weights. Clearly the best assay design will be that which gives the largest $Snw$ for the fewest assay animals. From this point of view, each animal may be thought of as contributing an amount ($w$) to the precision of the assay, and this amount depends on $Y$ ; that is on the actual number of reproductively intact cells given to the animal. Inspection of Appendix B, Table II, shows that $w$ is at its maximum when $Y = +0{\cdot}2$ : that is, when the mean number of intact cells per " dose " is about 1·6, and the response rate about 80 per cent.

The pooled control data analysed above will serve to illustrate this argument. We note that it is, in general, a well-designed assay, for no animals have been tested at " doses " with very low weighting coefficients. However, the group of 125 mice given a " dose " of one morphologically typical tumour cell per mouse contribute 179 to $Snw$ ; and only 54 mice tested at a " dose " of 4 cells would have contributed the same amount. Even here, then, a slight change in the design of the assay would have obtained the same precision with a saving of 71 mice.

If completely reliable advance information were available on the result to be expected from an assay, the assay would naturally be designed with only one group of assay animals. Every available animal would then be given a " dose " that was expected to contain on average 1·6 reproductively intact cells, and if all went well each assay animal would contribute the maximum to $Snw$. This, of course, is not a practical design for an assay, since with such a design inaccuracies in the advance information can have a disastrous effect on the precision of the assay, and even on the possibility of forming an estimate of log $E$ at all. For example, if the initial estimate were pessimistic by a factor of two, the " dose " given would contain on average 3·2 reproductively intact cells, and with a group of 20 animals there would be a 43 per cent risk that all animals would develop tumours.

Thus two factors affect the design of a practical assay ; the desirability of economy of assay animals, and the need for insurance against inaccurate advance information. If the advance information is unreliable, a wide range of " doses " must be used to provide insurance ; if the advance information is reliable, such insurance is merely wasteful.

In radiobiological work the proportion of cells treated in the same way which retain their reproductive integrity is expected to remain constant from one experiment to the next. If this condition is not fulfilled, then either the experiment has miscarried, or else any information that can be gleaned from it is not radiobiological. If it may be assumed that repeated assays will measure the same reproductively intact proportion, then the assays can be planned to give at each stage the appropriate amount of insurance.

Consider the case where the advance information has only the status of a wild guess. An assay design specifying groups of four animals, and " doses " of morphologically typical cells spaced by factors of eight, is appropriate. With four such groups of assay animals, the " doses " should be planned so that if the initial guess is correct the central two groups will receive $\frac{1}{4}$ and 2 intact cells per animal. Now if the initial guess is so wrong that the lowest " dose " contains more than 0·64 intact cells, then there will be a greater risk than 5 per cent that all the animals in the lowest group will succumb.

If all the animals in the lowest group do succumb, the assay will be uninformative, for even if some of the animals given higher doses escape, this will merely serve to cast suspicion on the execution of the assay, and it is more likely that every animal in the assay will succumb so that no estimate will be possible. Similarly if the initial guess is so wrong that the highest group receives less than 0·75 cells per animal, there will be a greater risk than 5 per cent that none of the animals in the highest group will succumb, again rendering the assay uninformative. If we wish to be at least 95 per cent certain that the assay will be informative, the initial guess must be within a range which extends approximately from 1/21st of the true value to 21 times it, and in this sense the insurance provided by this assay design extends from 21 times the initial guess to 1/21st of it. In the same way an assay design with five groups, each of four assay animals, and the " doses " again spaced by factors of eight, will give insurance from 1/59th of the initial guess to 59 times it: but here the central group should be given a " dose " which will contain 0·7 cells if the initial guess is correct.

Such preliminary assays pay a heavy price for insurance, since the average contribution to *Snw* of each assay animal will be less than 30 per cent of the maximum possible. Hence no very accurate estimate may be expected, and to increase the number of animals in each group above four would be imprudent. It will be more economical to use any extra animals in a subsequent, more efficient assay, which can have less insurance. The result of such a preliminary assay as these two will have 95 per cent confidence limits extending from one-third to thrice the estimate approximately.

From the results of such a preliminary assay, a second assay of cells treated in the same way can be planned. A design using three groups of animals, with " doses " separated by factors of four, and estimated (from the result of the preliminary assay) to contain $\frac{1}{4}$, 1 and 4 reproductively intact cells per " dose " would be appropriate. This design would have an entirely adequate amount of insurance, and the average contribution to *Snw* of the assay animals would be approximately 50 per cent of the maximum possible. Groups of less than five animals are undesirable, groups of more than ten are usually impracticable. If groups of six animals are used in this second assay, and if the result is compatible with that obtained in the preliminary assay, it will be possible to pool the two assays, and derive an estimate of the proportion of reproductively intact cells with confidence limits at about 53 per cent and 188 per cent of the estimate.

If further precision is desirable beyond this stage, it would be reasonable to reduce the amount of insurance still further. A design with three groups of assay animals given " doses " estimated on the basis of all available evidence to contain $\frac{1}{2}$, 1 and 2 reproductively intact cells would be expected to yield an average contribution to *Snw* from the assay animals of between 60 per cent and 75 per cent of the maximum possible. Designs with even less insurance may be regarded as too imprudent for most situations.

*The combination of estimates*

When two or more assays have been made of the proportion of reproductively intact cells among cells treated in the same way, the problem arises of combining the information from both assays. The two estimates could be formed, and a weighted mean obtained using the *Snw*'s as weights, but a better procedure is to pool the actual data, as if they were obtained in a single assay. If now the inconsistency $\chi^2$ is significantly large, this may mean that the component assays are incompatible, in which case the pooling would not be legitimate. The point can be investigated by analysing the component assays separately: if they are internally consistent (but incompatible) reasons should be sought for this. If, however, one or more of the component assays are themselves internally inconsistent, they may be rejected and an attempt made to pool the remainder.

### SUMMARY AND CONCLUSIONS

The statistical analysis of assays *in vivo* of the proportion of reproductively intact cells contained in tumour cell suspensions is discussed, and a method of analysis presented. This method of analysis, slightly modified from the method of Finney (1952), allows the internal consistency of the assay to be checked, and the standard error of the final estimate to be computed.

Applications to the design of such assays are made, distinguishing cases where advance information is unreliable, and the assay must allow for a wide range of possible outcomes, from cases where reliable advance information permits an assay design which will give higher precision from the minimum number of assay animals.

Thanks are due to Dr. Basil Shepstone for programming the tables of Appendix B for the Oxford University Digital Computer, to Dr. D. J. Finney, F.R.S., for helpful discussion, to Dr. J. R. Andrews for permission to use experimental results obtained jointly, and to Dr. Frank Ellis, Director of the Radiotherapy Department for enthusiastic encouragement.

R. J. B. is a Helen Hay Whitney Fellow in Radiobiology at Oxford University. These studies were aided by a grant from the British Empire Cancer Campaign.

### REFERENCES

Berry, R. J. and Andrews, J. R.—(1961) *Radiology*, **77**, 824.

Cruickshank, R.—(1960) 'Handbook of Bacteriology', 10th edition. Edinburgh (E. & S. Livingstone), p. 358.

Finney, D. J.—(1952) 'Statistical Method in Biological Assay'. London (Charles Griffin).

Hewitt, H. B.—(1958) *Brit. J. Cancer*, **12**, 378.

Pizzi, M.—(1950) *Hum. Biol.*, **22**, 151.

Reed, L. J. and Muench, H.—(1938) *Amer. J. Hyg.*, **27**, 493.

Silini, G. and Hornsey, S.—(1961) *Int. J. Radiat. Biol.*, **4**, 127.

### APPENDIX A

Reed and Muench (1938) proposed a rapid method for the statistical analysis of quantal data that has been extensively and uncritically applied to assays of the reproductive integrity of tumour cells. In this context, the only virtue of the Reed-Muench method is its disarming computational simplicity: its defects include an inappropriate theoretical background (see Finney (1952), for discussion), a total absence of validity tests and of estimates of precision, a tendency to bias, and (most serious of all) the compulsion to use an inefficient assay design.

The Reed-Muench method is a quick and simple one for the estimation of a 50 per cent effective dose, for use when a wide range of regularly spaced doses (extending from 0 per cent to 100 per cent effective) have been tested, each on the same number of assay animals. The numbers of animals responding to the different doses are summed from low dose to high ; and the numbers failing to respond are summed from high to low. The 50 per cent effective dose is estimated from these sums, either as the dose for which the two sums are equal, or by interpolation. The argument is that an animal which responds to a low dose would certainly have responded had the dose been higher ; one which fails to respond to a high dose would not have responded had the dose been smaller.

An example of the method is given in Appendix A, Table I, and it will be seen that the process of forming the sums involves the tacit assumptions that had a group of animals been given a higher " dose " than was actually tested, all would have succumbed, and that had a group been given a lower " dose ", all would have survived. An estimate of the $TD_{50}$ is formed by graphical interpolation, using the ratios

$$\frac{S(+)}{S(+) + S(-)} :$$

in this case it is 7500 cells.

Reed and Muench recommended interpolation in the logarithms of the doses, but in radiobiological work the custom has arisen of interpolating directly in the " doses ". This is somewhat less satisfactory than Reed and Muench's own procedure.

No way of assessing the precision of such a Reed-Muench estimate is known, except for the case of an underlying logistic distribution, where Pizzi (1950) has proposed a useful approximation. This approximation is not unreasonable, for the curve of $Q$ against log-dose differs little from the logistic form. No validity test (test of internal consistency) is available in the Reed-Muench method.

It may readily be seen that the Reed-Muench estimate is only unbiased if the chance of a response varies symmetrically about 50 per cent when plotted against dose. This can be demonstrated by applying the method to figures conforming to an asymmetric distribution. The sigmoid of a Poisson distribution is not symmetrical about $Q = 0{\cdot}5$ (i.e. about $x = \log_e 2$), whether $Q$ is plotted against $x$ or against the logarithm of $x$. Consequently the Reed-Muench method must introduce a bias into the estimate ; but when the number of animals per group is small, and the range of " doses " wide, this inherent bias is negligible.

When, however, the range of " doses " is narrow (as in the example given) a serious bias can arise from the use of the Reed-Muench method. This bias is small when the centre of the range of " doses " used is near to the true 50 per cent point ; but if the centre of the assay is moved away from the true 50 per cent point, the bias increases rapidly. In the example given, the bias entering in this way amounts to about 20 per cent.

If the Reed-Muench method of analysis is to be used, the design of the assay must be such as to avoid this serious source of bias. That is, the experimenter must plan his assay for a wide range of " doses ", so as to ensure as far as possible that the highest " dose " will produce 100 per cent responses, the lowest " dose "

0 per cent. This is, of course, quite contrary to the principles of economical assay design discussed above, for where advance information is available a more efficient assay design is possible. This compulsory waste of assay animals is the major defect of the Reed-Muench method.

*Appendix A, Table I.*—Anoxic mouse leukaemia cells P-388, after 3000 rads (250 kv)

| " Dose " | Mice responding (+) | Mice surviving (−) | $S(+)$ | $S(-)$ | $\frac{S(+)}{S(+)+S(-)}$ |
|---|---|---|---|---|---|
| 12,800 | 3 | 3 | 8 | 3 | $\frac{8}{11}$ |
| 6,400 | 3 | 3 | 5 | 6 | $\frac{5}{11}$ |
| 3,200 | 2 | 4 | 2 | 10 | $\frac{2}{12}$ |
| 1,600 | 0 | 6 | 0 | 16 | 0 |

APPENDIX B

*Appendix B, Table I*

| $Q$ | 0·00 | 0·01 | 0·02 | 0·03 | 0·04 | 0·05 | 0·06 | 0·07 | 0·08 | 0·09 |
|---|---|---|---|---|---|---|---|---|---|---|
| 0·0 | $-\infty$ | −2·00 | −1·70 | −1·52 | −1·39 | −1·29 | −1·21 | −1·14 | −1·08 | −1·03 |
| 0·1 | −0·98 | −0·93 | −0·89 | −0·86 | −0·82 | −0·79 | −0·76 | −0·73 | −0·70 | −0·68 |
| 0·2 | −0·65 | −0·63 | −0·61 | −0·58 | −0·56 | −0·54 | −0·52 | −0·50 | −0·48 | −0·47 |
| 0·3 | −0·45 | −0·43 | −0·41 | −0·40 | −0·38 | −0·37 | −0·35 | −0·34 | −0·32 | −0·31 |
| 0·4 | −0·29 | −0·28 | −0·26 | −0·25 | −0·24 | −0·22 | −0·21 | −0·20 | −0·18 | −0·17 |
| 0·5 | −0·16 | −0·15 | −0·13 | −0·12 | −0·11 | −0·10 | −0·09 | −0·07 | −0·06 | −0·05 |
| 0·6 | −0·04 | −0·03 | −0·01 | 0·00 | +0·01 | +0·02 | +0·03 | +0·05 | +0·06 | +0·07 |
| 0·7 | +0·08 | +0·09 | +0·11 | +0·12 | +0·13 | +0·14 | +0·15 | +0·17 | +0·18 | +0·19 |
| 0·8 | +0·21 | +0·22 | +0·23 | +0·25 | +0·26 | +0·28 | +0·29 | +0·31 | +0·33 | +0·34 |
| 0·9 | +0·36 | +0·38 | +0·40 | +0·43 | +0·45 | +0·48 | +0·51 | +0·55 | +0·60 | +0·66 |

This table gives values of $Y$, the logarithm of the average number of reproductively intact cells per " dose ". $Y$ is given for different values of $Q$, the theoretically expected proportion of animals that should respond to the corresponding " dose ". The function tabulated is :

$$Y = \log_{10} \{-\log_e (1 - Q)\}$$

*Note to Appendix B, Table II*

This table gives, for different values of $Y$, the corresponding values of $\phi_0$, $A$, $\phi_1$ and $w$. For the mathematical and logical details of the theory, the reader is referred to Finney (1952), who develops a method in which $Y$ is defined in terms of natural logarithms, and the *non-occurrence* of a tumour is taken formally as a " response ".

The method presented here, to which this table is appropriate, differs from Finney's method in its use of a $Y$ defined in terms of common logarithms, and in the use of the occurrence of a tumour as a response. These changes make the computations more convenient, but complicate the algebraic formulation of the functions tabulated. These are:

$$\phi_0 = \frac{1 - \exp\{10^Y\}}{\log_e 10 \times 10^Y}$$

$$A = \frac{\exp\{10^Y\}}{\log_e 10 \times 10^Y}$$

$$\phi_1 = \frac{1}{\log_e 10 \times 10^Y}$$

$$w = \frac{(\log_e 10)^2 \times 10^{2Y}}{\exp\{10^Y\} - 1}$$

The Oxford University Ferranti Mercury computer was used to compute the table, which it did in four minutes.

*Appendix B, Table II*

| $Y$ | $\phi_0$ | $A$ | $\phi_1$ | $w$ |
|---|---|---|---|---|
| 1·18 | −107,422·78686 | 107,422·81567 | 0·02869 | 0·00032 |
| 1·16 | −56,917·86120 | 56,917·89147 | 0·03005 | 0·00058 |
| 1·14 | −31,096·80798 | 31,096·83947 | 0·03146 | 0·00102 |
| 1·12 | −17,494·39636 | 17,494·42932 | 0·03294 | 0·00174 |
| 1·10 | −10,121·05267 | 10,121·08709 | 0·03450 | 0·00286 |
| 1·08 | −6,013·80093 | 6,013·83706 | 0·03612 | 0·00460 |
| 1·06 | −3,665·61503 | 3,665·65284 | 0·03783 | 0·00721 |
| 1·04 | −2,289·39292 | 2,289·43253 | 0·03961 | 0·01103 |
| 1·02 | −1,463·50002 | 1,463·54150 | 0·04147 | 0·01647 |
| 1·00 | −956·55226 | 956·59569 | 0·04343 | 0·02407 |
| 0·98 | −638·60567 | 638·65115 | 0·04548 | 0·03443 |
| 0·96 | −435·05966 | 435·10728 | 0·04762 | 0·04827 |
| 0·94 | −302·17519 | 302·22506 | 0·04986 | 0·06637 |
| 0·92 | −213·78726 | 213·83947 | 0·05221 | 0·08958 |
| 0·90 | −153·94062 | 153·99530 | 0·05467 | 0·11881 |
| 0·88 | −112·72593 | 112·78318 | 0·05725 | 0·15495 |
| 0·86 | −83·88009 | 83·94004 | 0·05995 | 0·19886 |
| 0·84 | −63·37786 | 63·44064 | 0·06277 | 0·25135 |
| 0·82 | −48·59056 | 48·65630 | 0·06573 | 0·31309 |
| 0·80 | −37·77515 | 37·84398 | 0·06883 | 0·38460 |
| 0·78 | −29·75886 | 29·83094 | 0·07207 | 0·46623 |
| 0·76 | −23·74157 | 23·81704 | 0·07547 | 0·55809 |
| 0·74 | −19·17004 | 19·24907 | 0·07903 | 0·66007 |
| 0·72 | −15·65685 | 15·73960 | 0·08275 | 0·77181 |
| 0·70 | −12·92740 | 13·01405 | 0·08665 | 0·89270 |
| 0·68 | −10·78473 | 10·87547 | 0·09074 | 1·02189 |
| 0·66 | −9·08605 | 9·18106 | 0·09501 | 1·15835 |
| 0·64 | −7·72666 | 7·82615 | 0·09949 | 1·30084 |
| 0·62 | −6·62907 | 6·73325 | 0·10418 | 1·44798 |
| 0·60 | −5·73533 | 5·84442 | 0·10909 | 1·59830 |
| 0·58 | −5·00171 | 5·11594 | 0·11423 | 1·75024 |
| 0·56 | −4·39491 | 4·51453 | 0·11961 | 1·90224 |
| 0·54 | −3·88936 | 4·01461 | 0·12525 | 2·05276 |
| 0·52 | −3·46525 | 3·59640 | 0·13115 | 2·20030 |
| 0·50 | −3·10713 | 3·24447 | 0·13734 | 2·34345 |

*Appendix B, Table II (continued)*

| $Y$ | $\phi_0$ | $A$ | $\phi_1$ | $w$ |
|---|---|---|---|---|
| 0·48 | −2·80287 | 2·94668 | 0·14381 | 2·48092 |
| 0·46 | −2·54283 | 2·69341 | 0·15059 | 2·61155 |
| 0·44 | −2·31934 | 2·47703 | 0·15768 | 2·73433 |
| 0·42 | −2·12626 | 2·29137 | 0·16511 | 2·84840 |
| 0·40 | −1·95859 | 2·13149 | 0·17290 | 2·95306 |
| 0·38 | −1·81231 | 1·99335 | 0·18104 | 3·04778 |
| 0·36 | −1·68410 | 1·87368 | 0·18958 | 3·13218 |
| 0·34 | −1·57126 | 1·76977 | 0·19851 | 3·20603 |
| 0·32 | −1·47153 | 1·67940 | 0·20787 | 3·26923 |
| 0·30 | −1·38306 | 1·60072 | 0·21766 | 3·32181 |
| 0·28 | −1·30428 | 1·53220 | 0·22792 | 3·36392 |
| 0·26 | −1·23389 | 1·47255 | 0·23866 | 3·39578 |
| 0·24 | −1·17079 | 1·42070 | 0·24991 | 3·41773 |
| 0·22 | −1·11404 | 1·37573 | 0·26169 | 3·43015 |
| 0·20 | −1·06287 | 1·33689 | 0·27402 | 3·43350 |
| 0·18 | −1·01658 | 1·30351 | 0·28694 | 3·42827 |
| 0·16 | −0·97460 | 1·27506 | 0·30046 | 3·41498 |
| 0·14 | −0·93644 | 1·25106 | 0·31462 | 3·39419 |
| 0·12 | −0·90166 | 1·23111 | 0·32945 | 3·36645 |
| 0·10 | −0·86989 | 1·21487 | 0 34497 | 3·33234 |
| 0·08 | −0·84082 | 1·20205 | 0·36123 | 3·29242 |
| 0·06 | −0·81414 | 1·19240 | 0·37825 | 3·24724 |
| 0·04 | −0·78963 | 1·18571 | 0·39608 | 3·19737 |
| 0·02 | −0·76706 | 1·18181 | 0·41475 | 3·14331 |
| 0·00 | −0·74624 | 1·18053 | 0·43429 | 3·08558 |
| −0·02 | −0·72701 | 1·18177 | 0·45476 | 3·02467 |
| −0·04 | −0·70921 | 1·18540 | 0·47619 | 2·96103 |
| −0·06 | −0·69271 | 1·19135 | 0·49864 | 2·89510 |
| −0·08 | −0·67740 | 1·19954 | 0·52214 | 2·82728 |
| −0·10 | −0·66318 | 1·20992 | 0·54674 | 2·75795 |
| −0·12 | −0·64994 | 1·22245 | 0·57251 | 2·68748 |
| −0·14 | −0·63760 | 1·23709 | 0·59949 | 2·61617 |
| −0·16 | −0·62610 | 1·25384 | 0·62775 | 2·54434 |
| −0·18 | −0·61535 | 1·27268 | 0·65733 | 2·47225 |
| −0·20 | −0·60531 | 1·29362 | 0·68831 | 2·40016 |
| −0·22 | −0·59591 | 1·31666 | 0·72075 | 2·32829 |
| −0·24 | −0·58710 | 1·34182 | 0·75472 | 2·25684 |
| −0·26 | −0·57885 | 1·36914 | 0·79029 | 2·18599 |
| −0·28 | −0·57111 | 1·39864 | 0·82753 | 2·11592 |
| −0·30 | −0·56383 | 1·43037 | 0·86653 | 2·04675 |
| −0·32 | −0·55700 | 1·46437 | 0·90737 | 1·97861 |
| −0·34 | −0·55057 | 1·50070 | 0·95013 | 1·91162 |
| −0·36 | −0·54452 | 1·53943 | 0·99491 | 1·84587 |
| −0·38 | −0·53882 | 1·58062 | 1·04180 | 1·78143 |
| −0·40 | −0·53345 | 1·62435 | 1·09090 | 1·71838 |
| −0·42 | −0·52839 | 1·67070 | 1·14231 | 1·65677 |
| −0·44 | −0·52361 | 1·71976 | 1·19615 | 1·59664 |
| −0·46 | −0·51910 | 1·77162 | 1·25252 | 1·53803 |
| −0·48 | −0·51484 | 1·82639 | 1·31155 | 1·48096 |
| −0·50 | −0·51081 | 1·88417 | 1·37336 | 1·42546 |
| −0·52 | −0·50700 | 1·94509 | 1·43808 | 1·37153 |
| −0·54 | −0·50340 | 2·00926 | 1·50586 | 1·31917 |
| −0·56 | −0·49999 | 2·07682 | 1·57683 | 1·26839 |
| −0·58 | −0·49677 | 2·14791 | 1·65114 | 1·21917 |
| −0·60 | −0·49371 | 2·22267 | 1·72896 | 1·17151 |

*Appendix B, Table II (continued)*

| $Y$ | $\phi_0$ | $A$ | $\phi_1$ | $w$ |
|---|---|---|---|---|
| −0·62 | −0·49081 | 2·30125 | 1·81044 | 1·12538 |
| −0·64 | −0·48807 | 2·38383 | 1·89576 | 1·08078 |
| −0·66 | −0·48546 | 2·47057 | 1·98511 | 1·03767 |
| −0·68 | −0·48299 | 2·56166 | 2·07866 | 0·99603 |
| −0·70 | −0·48065 | 2·65728 | 2·17663 | 0·95584 |
| −0·72 | −0·47843 | 2·75764 | 2·27921 | 0·91706 |
| −0·74 | −0·47632 | 2·86294 | 2·38663 | 0·87967 |
| −0·76 | −0·47431 | 2·97342 | 2·49910 | 0·84362 |
| −0·78 | −0·47241 | 3·08929 | 2·61688 | 0·80890 |
| −0·80 | −0·47060 | 3·21082 | 2·74021 | 0·77546 |
| −0·82 | −0·46888 | 3·33824 | 2·86936 | 0·74328 |
| −0·84 | −0·46725 | 3·47183 | 3·00458 | 0·71231 |
| −0·86 | −0·46570 | 3·61188 | 3·14619 | 0·68251 |
| −0·88 | −0·46422 | 3·75868 | 3·29446 | 0·65387 |
| −0·90 | −0·46282 | 3·91254 | 3·44972 | 0·62634 |
| −0·92 | −0·46148 | 4·07378 | 3·61230 | 0·59988 |
| −0·94 | −0·46021 | 4·24276 | 3·78255 | 0·57446 |
| −0·96 | −0·45900 | 4·41981 | 3·96081 | 0·55005 |
| −0·98 | −0·45785 | 4·60533 | 4·14748 | 0·52662 |
| −1·00 | −0·45675 | 4·79970 | 4·34295 | 0·50412 |
| −1·02 | −0·45571 | 5·00333 | 4·54762 | 0·48254 |
| −1·04 | −0·45471 | 5·21666 | 4·76194 | 0·46182 |
| −1·06 | −0·45377 | 5·44014 | 4·98637 | 0·44196 |
| −1·08 | −0·45287 | 5·67424 | 5·22137 | 0·42291 |
| −1·10 | −0·45201 | 5·91945 | 5·46744 | 0·40464 |
| −1·12 | −0·45119 | 6·17631 | 5·72512 | 0·38713 |
| −1·14 | −0·45041 | 6·44535 | 5·99493 | 0·37034 |
| −1·16 | −0·44967 | 6·72714 | 6·27747 | 0·35426 |
| −1·18 | −0·44896 | 7·02228 | 6·57331 | 0·33885 |
| −1·20 | −0·44829 | 7·33139 | 6·88310 | 0·32408 |
| −1·22 | −0·44765 | 7·65514 | 7·20750 | 0·30994 |
| −1·24 | −0·44703 | 7·99421 | 7·54717 | 0·29640 |
| −1·26 | −0·44645 | 8·34931 | 7·90286 | 0·28343 |
| −1·28 | −0·44589 | 8·72121 | 8·27531 | 0·27101 |
| −1·30 | −0·44536 | 9·11068 | 8·66532 | 0·25912 |
| −1·32 | −0·44486 | 9·51856 | 9·07370 | 0·24774 |
| −1·34 | −0·44437 | 9·94570 | 9·50133 | 0·23685 |
| −1·36 | −0·44391 | 10·39303 | 9·94911 | 0·22642 |
| −1·38 | −0·44347 | 10·86148 | 10·41800 | 0·21645 |
| −1·40 | −0·44306 | 11·35204 | 10·90899 | 0·20690 |
| −1·42 | −0·44266 | 11·86577 | 11·42311 | 0·19776 |
| −1·44 | −0·44227 | 12·40374 | 11·96147 | 0·18903 |
| −1·46 | −0·44191 | 12·96710 | 12·52519 | 0·18067 |
| −1·48 | −0·44156 | 13·55705 | 13·11549 | 0·17267 |
| −1·50 | −0·44123 | 14·17484 | 13·73360 | 0·16502 |
| −1·52 | −0·44092 | 14·82176 | 14·38085 | 0·15771 |
| −1·54 | −0·44062 | 15·49921 | 15·05859 | 0·15071 |
| −1·56 | −0·44033 | 16·20861 | 15·76828 | 0·14402 |
| −1·58 | −0·44006 | 16·95148 | 16·51142 | 0·13763 |
| −1·60 | −0·43979 | 17·72937 | 17·28958 | 0·13151 |
| −1·62 | −0·43955 | 18·54396 | 18·10441 | 0·12566 |
| −1·64 | −0·43931 | 19·39696 | 18·95765 | 0·12007 |
| −1·66 | −0·43908 | 20·29017 | 19·85109 | 0·11473 |
| −1·68 | −0·43886 | 21·22551 | 20·78665 | 0·10962 |
| −1·70 | −0·43866 | 22·20495 | 21·76629 | 0·10473 |

*Appendix B, Table II (continued)*

| $Y$ | $\phi_0$ | $A$ | $\phi_1$ | $w$ |
|---|---|---|---|---|
| −1·72 | −0·43846 | 23·23057 | 22·79211 | 0·10007 |
| −1·74 | −0·43827 | 24·30454 | 23·86627 | 0·09560 |
| −1·76 | −0·43809 | 25·42914 | 24·99105 | 0·09134 |
| −1·78 | −0·43792 | 26·60676 | 26·16884 | 0·08726 |
| −1·80 | −0·43775 | 27·83989 | 27·40214 | 0·08337 |
| −1·82 | −0·43760 | 29·13116 | 28·69356 | 0·07964 |
| −1·84 | −0·43745 | 30·48330 | 30·04585 | 0·07608 |
| −1·86 | −0·43731 | 31·89917 | 31·46187 | 0·07268 |
| −1·88 | −0·43717 | 33·38179 | 32·94462 | 0·06943 |
| −1·90 | −0·43704 | 34·93429 | 34·49725 | 0·06633 |
| −1·92 | −0·43692 | 36·55997 | 36·12306 | 0·06336 |
| −1·94 | −0·43680 | 38·26228 | 37·82548 | 0·06053 |
| −1·96 | −0·43668 | 40·04483 | 39·60814 | 0·05782 |
| −1·98 | −0·43658 | 41·91140 | 41·47482 | 0·05523 |
| −2·00 | −0·43647 | 43·86594 | 43·42947 | 0·05275 |
| −2·02 | −0·43637 | 45·91261 | 45·47623 | 0·05039 |
| −2·04 | −0·43628 | 48·05574 | 47·61946 | 0·04813 |
| −2·06 | −0·43619 | 50·29989 | 49·86370 | 0·04598 |
| −2·08 | −0·43611 | 52·64981 | 52·21370 | 0·04392 |
| −2·10 | −0·43602 | 55·11048 | 54·67446 | 0·04195 |
| −2·12 | −0·43595 | 57·68713 | 57·25119 | 0·04007 |
| −2·14 | −0·43587 | 60·38522 | 59·94935 | 0·03827 |
| −2·16 | −0·43580 | 63·21048 | 62·77468 | 0·03655 |
| −2·18 | −0·43573 | 66·16889 | 65·73315 | 0·03491 |
| −2·20 | −0·43567 | 69·26673 | 68·83106 | 0·03335 |
| −2·22 | −0·43561 | 72·51057 | 72·07497 | 0·03185 |
| −2·24 | −0·43555 | 75·90730 | 75·47176 | 0·03042 |
| −2·26 | −0·43549 | 79·46412 | 79·02863 | 0·02906 |
| −2·28 | −0·43544 | 83·18857 | 82·75313 | 0·02775 |
| −2·30 | −0·43538 | 87·08855 | 86·65317 | 0·02651 |
| −2·32 | −0·43534 | 91·17234 | 90·73701 | 0·02532 |
| −2·34 | −0·43529 | 95·44859 | 95·01331 | 0·02418 |
| −2·36 | −0·43524 | 99·92639 | 99·49114 | 0·02309 |
| −2·38 | −0·43520 | 104·61522 | 104·18002 | 0·02206 |
| −2·40 | −0·43516 | 109·52503 | 109·08987 | 0·02107 |
| −2·42 | −0·43512 | 114·66624 | 114·23112 | 0·02012 |
| −2·44 | −0·43508 | 120·04975 | 119·61466 | 0·01922 |
| −2·46 | −0·43505 | 125·68697 | 125·25192 | 0·01835 |
| −2·48 | −0·43501 | 131·58988 | 131·15486 | 0·01753 |
| −2·50 | −0·43498 | 137·77099 | 137·33600 | 0·01674 |
| −2·52 | −0·43495 | 144·24340 | 143·80845 | 0·01599 |
| −2·54 | −0·43492 | 151·02085 | 150·58593 | 0·01527 |
| −2·56 | −0·43489 | 158·11772 | 157·68282 | 0·01458 |
| −2·58 | −0·43487 | 165·54905 | 165·11418 | 0·01393 |
| −2·60 | −0·43484 | 173·33062 | 172·89578 | 0·01330 |
| −2·62 | −0·43482 | 181·47892 | 181·04410 | 0·01270 |
| −2·64 | −0·43479 | 190·01124 | 189·57645 | 0·01213 |
| −2·66 | −0·43477 | 198·94567 | 198·51090 | 0·01159 |
| −2·68 | −0·43475 | 208·30118 | 207·86643 | 0·01107 |
| −2·70 | −0·43473 | 218·09761 | 217·66288 | 0·01057 |
| −2·72 | −0·43471 | 228·35572 | 227·92101 | 0·01009 |
| −2·74 | −0·43469 | 239·09728 | 238·66259 | 0·00964 |
| −2·76 | −0·43467 | 250·34509 | 249·91041 | 0·00921 |
| −2·78 | −0·43465 | 262·12297 | 261·68832 | 0·00879 |
| −2·80 | −0·43464 | 274·45594 | 274·02130 | 0·00840 |